# Perforation of the Intestines by Worms

**Published:** 1863-01

**Authors:** J. F. Norton

**Affiliations:** Fort Edward, N. Y.


For the Buffalo Medical and Surgical Journal.

ART. II.—*Perforation of the Intestines by Worms*—By J. F. Norton.

It seems yet to be a question of much doubt among most authors, whether worms that are found in the intestines, and particularly the lumbricus, which it is claimed by some, are peculiarly organized for that purpose, are capable of perforating the intestinal coats. The question is certainly one of interest, and of late has been made the subject of much medico-legal discussion. I therefore deem it my duty to throw so much light upon the subject as experience will allow. Some three cases in all, have come under my observation during the last fifteen years, which by many might be called perforation by worms, It would be quite unnecessary for me to detail each successive case, as the last is of recent date, and characterizes the others in all essential particulars.

I was hastily summoned to the residence of a farmer, living in the country. I had just previously visited the mother of the family, who lay ill at the time. I entered the house expecting to witness some untoward change in the patient, but in this I was no less disappointed than surprised, for instead of the mother I was conducted to the bedside of a lad some twelve years of age, the very boy who called me to his mother in the morning, and he, too, in a dying condition. He lay upon his back, with his legs drawn up, undoubtedly to relieve the tension of the abdominal muscles; skin pale, cold and bedewed with a clammy sweat, his eyes were unusually bright and anxious, with an expressive and solicitous countenance; pulse low, fluctuating, and almost indistinct; breathing hurried and laborious; when questioned he answered hastily; when asked where he suffered from pain, he placed his hand upon the umbilical and right iliac region. In turning down the bedclothes I found the whole abdominal region distended, and intolerant to pressure; his thirst was insatiable; tympanitic symptoms soon presented, with *singultus* and *nausea.* I but partially succeeded in relieving these distressing symptoms, when death closed the scene, having lived about fourteen hours from the attack. I examined the body in the morning following; it was greatly distended with air, fluids, &c., rendering the post mortem extremely unpleasant. I displaced so much of the water as was necessary, and proceeded to examine the small intestines, having a strong suspicion of the nature of the case. Near the lower portion of the ileum my attention was arrested by its blackened appearance. My expectations were now fully realized, when I discovered a large lumbricus, partially within the peritoneal cavity, and partially within the intestines; this black looking portion I tied, and detached some six inches in length. The lower part of this piece of gut, and below the perforation in which the worm was suspended, contained a hard, globular mass, about the size of a hen's egg, which proved to be a knot of worms, and so compact as to be difficult to separate, and completely blocking the channel. This portion of intestine was of a dark blue and velvety appearance, and occasionally interspersed with spots of a yellow red. The perforation occupied the center of one of these ulcerous spots; the base of this sloughing ulcer was near the size of a half dollar, while the apperture through the serous coat was about the size of a dime piece. The mucous, cellular and muscular coats, were badly disorganized, leaving the thin serous coat denuded and bare in several places.

To conclude, I must say that my experience, both practically and theoretically, induce me to believe that perforation of the intestinal coats by worms, is extremely doubtful, if indeed, there exists one well authenticated case, and is it not much more reasonable to adopt the more rational theory, that an accumulation of lumbriæ, in certain portions of the intestinal canal, will give rise to an irritation, which if not removed, must terminate in inflammation, ulceration and sloughing of its tunics, and the worms, with the contents of the bowels escape into the peritoneal cavity!

Fort Edward, N. Y., December, 1862.

